# A furano–*ortho*–vanillin conjugate for fluorogenic ratiometric and selective Zn^2+^ sensing: theoretical insights and biological studies

**DOI:** 10.1039/d5ra09973k

**Published:** 2026-06-01

**Authors:** Priyanka Avala, Shinziya H., Avijit Kumar Das, Gopal Ch. Das, Tilak Raj Maity, Aveek Samanta, Malay Dolai

**Affiliations:** a Department of Chemistry, Christ University Hosur Road Bangalore Karnataka 560029 India avijitkumar.das@christuniversity.in sanjuavi.das@gmail.com; b Centre for Renewable Energy and Environmental Sustainability, Christ University Karnataka 560029 India; c Department of Chemistry, Prabhat Kumar College Purba Medinipur 721404 West Bengal India E-mail-dolaimalay@yahoo.in; d Department of Biotechnology, Haldia Institute of Technology Purba Medinipur 721657 Haldia West Bengal India; e Department of Botany, Prabhat Kumar College Purba Medinipur Contai 721404 W.B. India

## Abstract

A fluorescent sensor BFC was designed and synthesized in order to identify the selective fluorescence recognition of Zn^2+^ in semi-aqueous environments. BFC notably displayed ratiometric red-shifted, twelve-fold fluorescence “turn-on” enhancement when exposed to Zn^2+^ at 490 nm with an isoemission point at 440 nm and a detection limit of 0.65 µM, which is considerably below the WHO (World Health Organisation) recommended value of Zn^2+.^ The binding constant of BFC with Zn^2+^ was determined as 4 × 10^4^ M^−1^. The fluorescence enhancement of BFC in the presence of Zn^2+^ is attributed to the enhancement of charge transfer leading to high fluorescence *via* the CHEF mechanism. The binding interaction between BFC and Zn^2+^ was elucidated using UV-vis and fluorescence spectroscopy, supported by Job's plot analysis and theoretical insights from DFT calculations. For biological applications, BFC has been employed in plant-based cell imaging to monitor Zn^2+^ accumulation in *Lathyrus sativus* L. (grass pea). Overall, BFC presents a simple, effective, and promising fluorescent probe for ratiometric Zn^2+^ detection in diverse fields such as a versatile tool for Zn^2+^ detection in different applications like detoxification and marker molecules for bioabsorption.

## Abstract 2

A fluorescent sensor BFC was designed and synthesized in order to identify the selective fluorescence recognition of Zn^2+^ in semi-aqueous environments.

## Graphical abstract

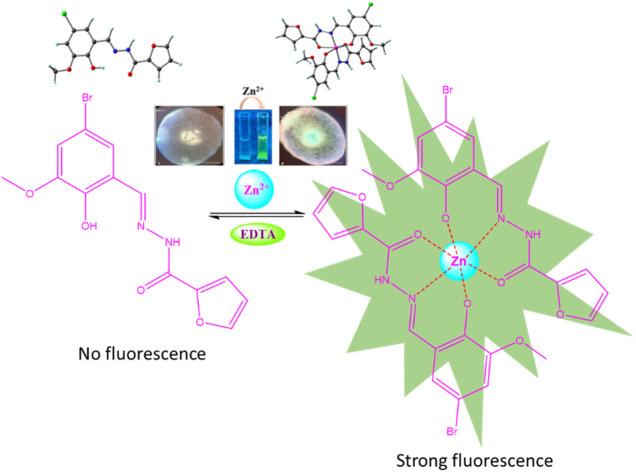

## 1. Introduction

Metal ions play vital roles in both chemical and biotic systems owing to their distinct binding affinities and ligand exchange dynamics, which confer remarkable selectivity in diverse environments. Among these, zinc ions (Zn^2+^) have received particular attention due to their essential physiological and environmental significance. Biologically, zinc is indispensable for numerous processes, including endocrine regulation, thyroid hormone metabolism^1,2^ male reproductive health^3,4^ neurological development^5^ and retinal protection.^6^ Moreover, adequate zinc levels help prevent age-related macular degeneration and maintain immune function.^7,8^ In contrast, zinc deficiency has been linked to various health disorders such as impaired growth, immune dysfunction, delayed maturation, sensory impairments, adverse pregnancy outcomes, and recurrent infections.^9–12^ Even marginal deficiencies can negatively affect clinical, biochemical, and immunological parameters.^13–16^ Parallel to its biological relevance, zinc pollution resulting from industrial activities such as mining, galvanizing, and battery manufacturing has raised environmental concerns. Consequently, the sensitive and selective detection of Zn^2+^ is of great importance for both environmental monitoring and biomedical diagnostics. Although several analytical techniques—including atomic absorption spectrometry,^17^ ion-selective membrane electrodes,^18^ and inductively coupled plasma-mass spectrometry^19^-have been developed for zinc quantification, these methods typically require costly instrumentation, skilled personnel, and laborious sample preparation. In contrast, fluorescent chemosensors offer a highly promising alternative due to their operational simplicity, rapid response, and excellent selectivity and sensitivity in solution-based detection.^20,21^

Designing fluorescent probes for Zn^2+^, however, remains a formidable challenge. Owing to the filled d^10^ electronic configuration of Zn^2+^, it does not exhibit d–d transitions and often fails to interact strongly with many conventional sensing ligands.^22–27^ Effective probe design therefore demands careful integration of a coordinating probes with a fluorescent unit capable of transducing the binding event into a measurable fluorescence signal.^28,29^ In this respect, photoinduced electron transfer (PET), aggregation-induced emission (AIE) and activity-based sensing mechanism have been extensively employed for the detection landscape. Nonetheless, ICT processes are often restricted to specific fluorophores, and FRET typically requires complex architectures involving two chromophores, which limit general applicability.^30–34^ The most effective strategy for overcoming these issues and ensuring reliability involves the use of ratiometric approaches. Ratiometric fluorescent probes operate by exhibiting analyte-induced variations in the emission intensities of two or more distinct bands. The ratio of fluorescence intensities at these wavelengths is directly proportional to the concentration of the target analyte, providing an intrinsic self-calibration mechanism. This internal referencing significantly enhances sensitivity and accuracy, allowing reliable quantitative analysis. As a result, ratiometric sensing effectively minimizes the influence of environmental fluctuations and instrumental variations, thereby reducing measurement ambiguities.^35–37^

In this context, we have developed a multifunctional Schiff base probe, (*E*)-*N*′-(5-bromo-2-hydroxy-3-methoxybenzylidene)furan-2-carbohydrazide (BFC), for the ratiometric fluorescence detection of Zn^2+^ ion. The probe was synthesized *via* condensation between 5-bromo-2-hydroxy-3-methoxybenzaldehyde and furan-2-carbohydrazide. Schiff based fluorescent probes have been widely explored for Zn^2+^ detection because of their simple synthesis and strong metal binding capability.^38^ However, many reported systems suffer from limited fluorescence enhancement due to active non-radiative decay pathways associated with flexible –C=N– isomerization. To overcome this limitation, our probe was strategically designed to exploit a Zn^2+^ induced chelation mechanism that rigidifies the molecular framework upon binding. This structural locking suppresses non-radiative decay, strengthens the internal charge-transfer process, and effectively activates a pronounced chelation-enhanced fluorescence (CHEF) response. By integrating a hydroxy-imine binding site capable of deprotonation and stable metal coordination, the probe achieves intensified absorbance and red shifted ratiometric fluorescence that distinguish it from conventional Schiff base as reported Zn^2+^ sensors (Table S1). Comprehensive characterization of BFC was performed using NMR, mass spectra and IR Spectra (SI, Fig. S4–S7). Density functional theory (DFT) and biological studies were further employed to elucidate its sensing behaviour and validate its practical applicability.

## 2 Results and discussions

### 2.1 Synthetic procedure and characterization of BFC

**Scheme 1:**
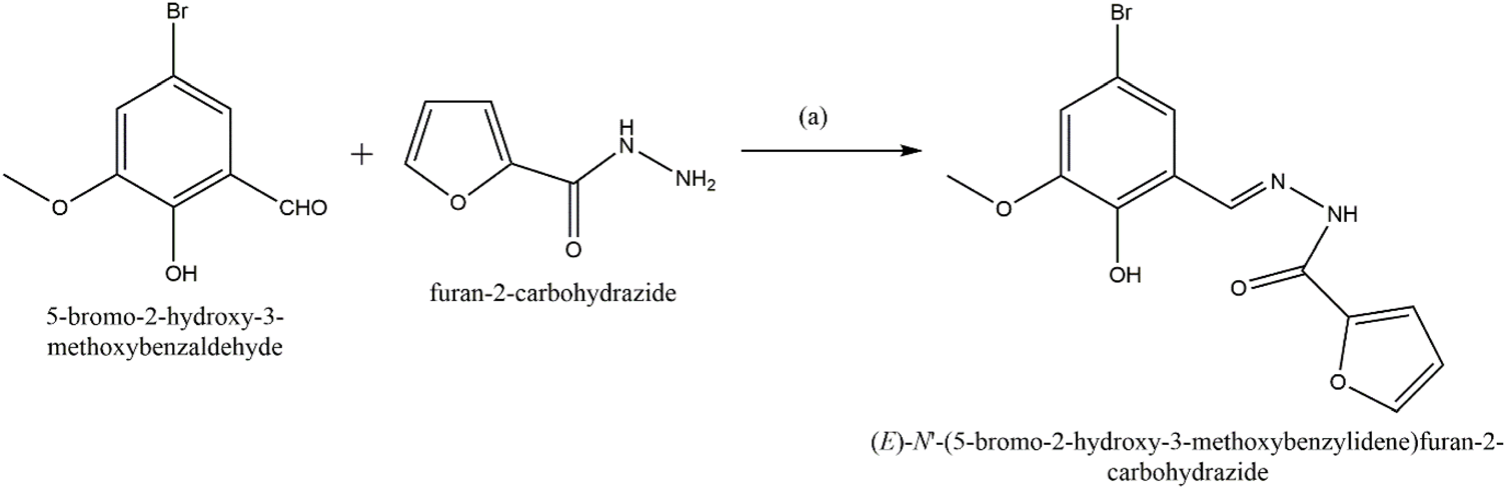
Synthesis of BFC: (a) EtOH, rt, 2 h.

The solution of 5-bromo-3-methoxy-2 hydroxy benzaldehyde (339 mg, 1 mmol) in ethanol and solution of 2-furoic-hydrazide (126 mg, 1 mmol) in ethanol was mixed together on stirring for 2 hours. After completion the reaction by TLC monitoring, a light-yellow precipitate was separated out, which was filtered and washed with cold methanol and dried to recover a pale-yellow powder compound as BFC (Scheme 1). ^1^H NMR (DMSO-*d*_6_, 400 MHz): *δ* (ppm): 12.14 (s, 1H, –OH), 10.72 (s, 1H, –NH), 8.63 (s, 1H, =CH), 7.96 (s, 1H), 7.35 (d, 2H), 7.16 (s, 1H), 6.72 (s, 1H), 3.84 (s, 3H, –CH_3_). ^13^C NMR (DMSO-*d*_6_, 100 MHz): *δ* (ppm): 154.52, 149.63, 146.60, 145.96, 121.95, 121.45, 116.47, 115.79, 112.63, 110.65, 56.76. Mass (*m*/*z*, %): M^+^ calculated for chemical formula: C_13_H_11_BrN_2_O_4_ is 337.9902; found: 339.3256 (M + H)^+^. FTIR (cm^−1^): 2900–3744 (broad, –OH, –NH), 2260, 1635 (–CO), 1441, 1374, 1204, 1035 (C–O–C), 911, 634.

### 2.2 Binding study with Zn^2+^

To study the binding behaviour of BFC with Zn^2+^, absorption and emission studies were carried out in CH_3_CN-HEPES buffer (9 : 1, v/v, pH = 7.4). On performing the UV-vis studies, BFC displayed absorption peaks at 310 and 340 nm. With the stepwise addition of Zn^2+^, there are emergence of new peaks at 325 nm and 400 nm with three isosbestic points centred at 318 nm, 348 nm, 360 nm. For free BFC and BFC–Zn^2+^ complex, the molar extinction coefficient at 400 nm was determined to be 4.45 × 10^3^ M^−1^ cm^−1^ and 9.05 ×10^2^ M^−1^ cm^−1^, respectively.

**Fig. 1:**
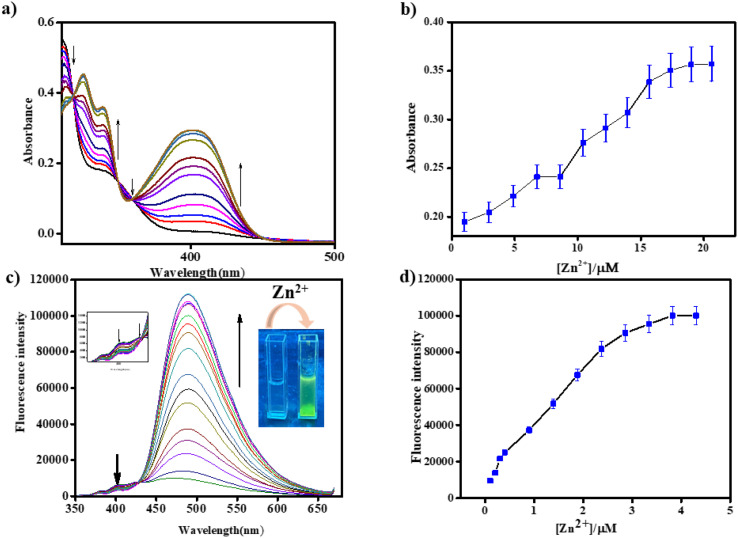
(a) and (c) UV-vis and fluorescence titration of BFC (*c* = 2 × 10^−5^ M) upon addition of Zn^2+^ (*c* = 2 × 10^−4^ M, 10 equiv.) (enlarged view of the isoemission point). (b) and (d) changes of concentration *vs.* intensity of BFC for Zn^2+^ in UV-vis (*λ*_abs_ = 340 nm) and fluorescence spectra (*λ*_em_ = 490 nm) respectively.

In the fluorescence studies, the free ligand BFC displayed weak emission maxima at 400 nm and 490 nm upon excitation at 340 nm, with a quantum yield (*Φ*) of 0.74. But the addition of Zn^2+^, a bathochromic shift was observed along with a red shifted enhanced ratiometric emission intensity enhancement at 490 nm (Δ*λ* = 90 nm) with a decrease of the emission signal at 400 nm with an isoemission point at 440 nm (Fig. 1c). The fluorescence intensity increased nearly 12-fold, with the quantum yield (*Φ*) rising to 0.82. A distinct fluorescence color change from colorless to green was observed (Fig. 1c, inset: cuvette image). Both the absorbance and fluorescence intensity of BFC increased progressively with increasing Zn^2+^ concentration, eventually reaching saturation at higher concentrations. This behaviour is consistent with the CHEF mechanism, in which Zn^2+^ coordination restricts non-radiative decay pathways and enhances the emission from the longer-wavelength band. As a result, fluorescence at 490 nm is amplified, while the shorter-wavelength emission at 400 nm is suppressed. The limit of detection of BFC toward Zn^2+^ was determined to be 0.65 µM by fluorometric analysis (Fig. S2).^39^ From the fluorescence titration experiment, the association constant (*K*_a_) of BFC with Zn^2+^ was estimated as 4 × 10^4^ M^−1^ (error <10%) (Fig. S1).^40^ From the time-dependent fluorescence changes of BFC upon the incremental addition of Zn^2+^, the binding rate constant was determined to be 7.86 × 10^2^ s^−1^ using a first-order kinetic model, confirming the fast response of BFC toward zinc ions (Fig. S8). Moreover, pH-dependent studies revealed that in the absence of Zn^2+^, BFC exhibits relatively weak fluorescence across the entire pH range of 2–14. In contrast, upon addition of Zn^2+^, a pronounced enhancement in fluorescence intensity is observed, particularly around physiological pH. Notably, BFC shows very weak emission under strongly acidic or highly basic conditions, whereas a strong Zn^2+^-induced fluorescence response is evident near neutral pH. Under strongly acidic conditions, protonation of the donor atoms like N and O, which inhibits Zn^2+^ coordination and promotes non-radiative decay, leading to weak fluorescence. Under highly basic conditions, hydroxide competition and possible Zn(OH)_2_ formation reduce effective binding with BFC. These results demonstrate that BFC operates most efficiently as a zinc sensor under biologically relevant conditions (Fig. S9).

### 2.3 Interference study

**Fig. 2:**
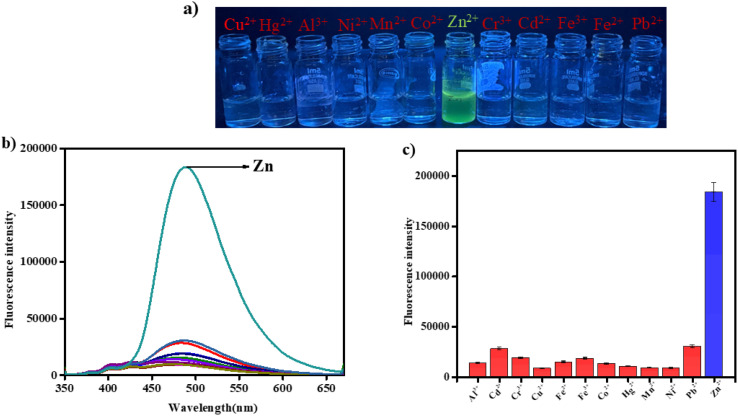
(a) Fluorescence colour response of probe BFC with different competing metal ions. (b) The emission spectra and (c) bar representation of BFC after adding different interfering metal ions (*c* = 2 × 10^−4^ M, 10 equiv.) in CH_3_CN/HEPES buffer solution (9/1, v/v, pH = 7.4) (*λ*_em_ = 490 nm).

A fluorescence experiments have been performed to assess the selectivity of BFC towards Zn^2+^ among different competing metal ions, including Cu^2+^, Al^3+^, Ni^2+^, Mn^2+^, Co^2+^, Cd^2+^, Fe^2+^, Fe^3+^, Pb^2+^, Hg^2+^, Cr^3+^ in CH_3_CN/HEPES buffer solution (9 : 1, v/v, pH 7.4). The results revealed that only Zn^2+^ induced a pronounced enhancement in the fluorescence intensity of BFC at 490 nm, but no discernible change was seen with other metal ions (Fig. 2a and b). The corresponding bar graph further highlights the selective fluorescent response of BFC, where the blue bar representing Zn^2+^ shows the maximum signal, while the red bars corresponding to the remaining metal ions indicate negligible interaction and minimal enhancement in fluorescence (Fig. 2c).

### 2.4 Competition study

**Fig. 3:**
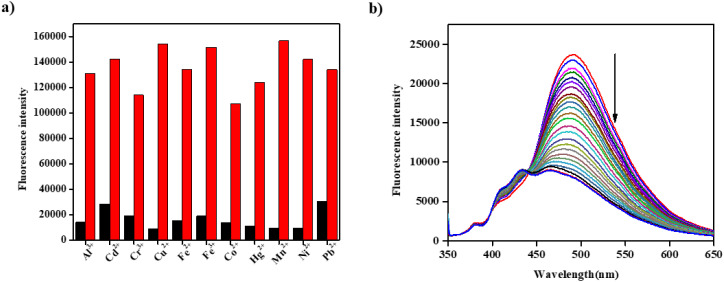
(a) The fluorescence intensity of BFC (*c* = 2 × 10^−5^ M) with 10 equiv. of different metal ions (*c* = 2 × 10^−4^ M) (black bars); the fluorescence changes of BFC with 10 equiv. of other metal ions in the presence of 10 equiv. of Zn^2+^ (red bars). (b) Fluorescence changes of BFC + Zn^2+^ complex solution (*c* = 2 × 10^−5^ M) on gradual addition of EDTA (*c* = 2 × 10^−4^ M).

Fluorometric analysis was conducted to examine the impact of competing metal ions on the binding affinity of BFC for Zn^2+^, thereby assessing its selectivity. A cross-contamination study was conducted using 10 equiv. of Zn^2+^ in the presence of various competing metal ions. The results showed that the fluorescence response induced by Zn^2+^ binding (red bars) remained essentially unchanged in the presence of other metal ions, while these potential interferents (black bars) had no significant effect on BFC. These observations confirm that the BFC receptor demonstrates outstanding selectivity and sensitivity toward Zn^2+^ (Fig. 3a).

### 2.5 Reversibility test

The binding behaviour of BFC with Zn^2+^ was further examined using fluorescence titration experiments. Upon incremental addition of EDTA (0–2.0 equivalents) to the BFC–Zn^2+^ complex, the strong chelation of Zn^2+^ with EDTA displaced it from the BFC binding site, leading to a progressive decrease in fluorescence intensity (Fig. 3b). Which produced a distinct reversible on–off switching of fluorescence between free BFC and the BFC–Zn^2+^ complex. These results confirm the reversible coordination of Zn^2+^ with BFC.

### 2.6 Mechanism of optical response and probable binding mode in the solution phase

**Scheme 2:**
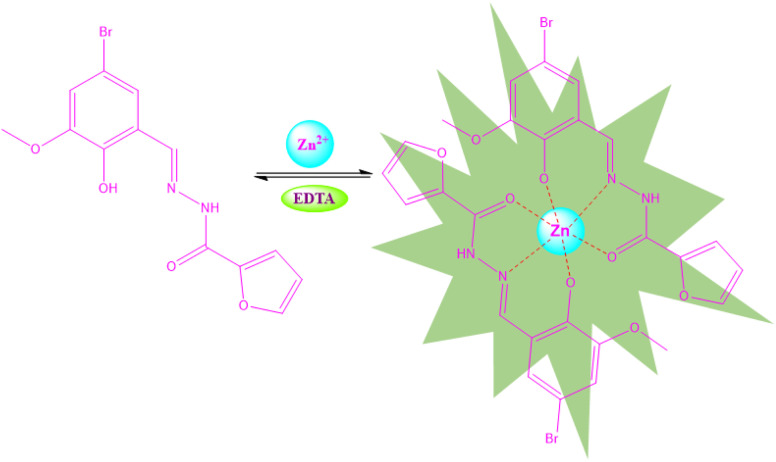
Probable binding mode in solution phase.

The binding pathways and interaction of BFC with Zn^2+^ have been further demonstrated by UV-vis, fluorescence and DFT analysis. In the absence of metal ions, BFC shows weak absorbance and emission, attributed to the conjugated imine (–C=N–) bond and the surrounding electron system, which facilitates non-radiative decay pathways such as structural isomerization *via* the –C=N– bond.^41^ In contrast, the addition of Zn^2+^ leads to the appearance of an isosbestic point in the UV-vis spectra and ratiometric response with an isoemission point in the fluorescence spectra, indicating strong metal–ligand interactions and the formation of a stable BFC–Zn^2+^ complex. These spectral features indicate a direct, stoichiometric transformation between the free ligand BFC and Zn^2+^ bound forms of the sensor, confirming the ratiometric response. The observed increase in absorbance along with a red-shifted ratiometric fluorescence enhancement arises from an intensified charge transfer (CT) process between BFC and Zn^2+^ by the deprotonation of hydroxy group BFC, which suppresses –C=N– isomerization and activates a chelation-enhanced fluorescence (CHEF) effect by the formation of stable BFC–Zn complex.^42^ Upon coordination with Zn^2+^, BFC adopts a hexadentate binding mode through two ligand centers, involving hydroxyl oxygen, hydrazide oxygen, and imine nitrogen atoms from both ligands (Scheme 2). This chelation restricts non-radiative decay pathways (on-state), thereby amplifying the fluorescence intensity *via* the CHEF mechanism. The 2 : 1 binding stoichiometry between ligand BFC with Zn^2+^ obtained from Job's plot analysis (Fig. S3) and theoretical calculations corroborates this assignment.

### 2.7 Theoretical study

**Fig. 4:**
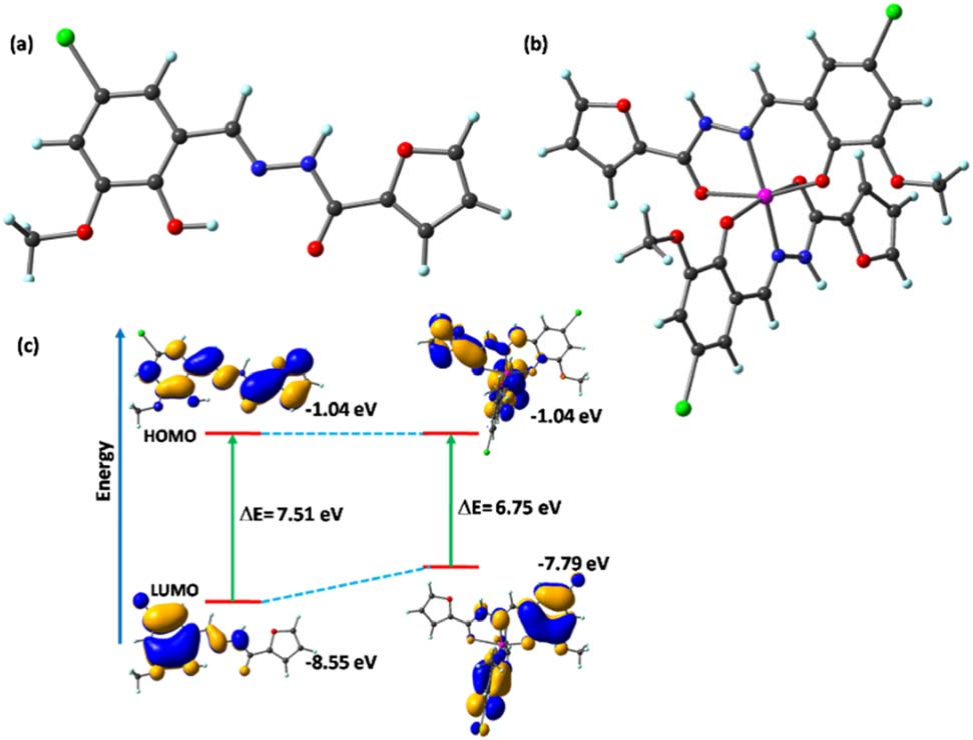
(a) Geometry-optimized molecular structure of (a) BFC and (b) the BFC–Zn^2+^ complex. (c) Frontier molecular orbitals and the corresponding energy gaps of BFC and the BFC–Zn^2+^ complex.

To demonstrate the binding pattern of BFC with Zn^2+^, structural optimizations of BFC and BFC–Zn^2+^ complex were performed using density functional theory (DFT) at the B3LYP level (Fig. 4a and b). In the optimized structure of BFC–Zn^2+^ complex, zinc bound with two BFC ligands with six co-ordination with the binding donor centres nitrogen and oxygen. The spatial distribution of the electron cloud and the energies of the frontier molecular orbitals (FMOs)—specifically the highest occupied molecular orbital (HOMO) and the lowest unoccupied molecular orbital (LUMO)—were investigated for both the BFC and the BFC–Zn^2+^ complex. For BFC, the HOMO–LUMO energy gap was calculated to be 7.51 eV, with HOMO and LUMO energies of −8.55 eV and −1.04 eV, respectively. In comparison, BFC–Zn^2+^ complex showed a lower energy gap of 6.75 eV, with HOMO and LUMO energies of −7.79 eV and −1.04 eV (Fig. 4c). The decrease of energy gap between HOMO and LUMO indicates the stabilization of BFC–Zn^2+^ complex, supports the thermodynamic feasibility of the conversion.

### 2.8 Biological applications

#### 2.8.1 Antioxidant assay and bioimaging study

**Fig. 5:**
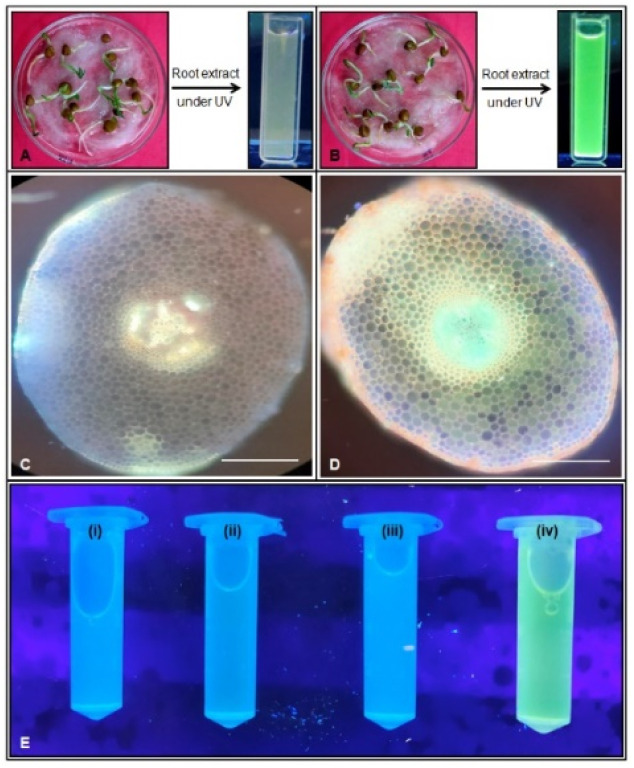
Estimation of Zn^2+^ in grass pea roots. (A) Fluorescence of root extract under UV light and seedlings treated with distilled water (control). (B) Fluorescence of root extract under UV light and seedlings treated Zn^2+^ followed by BFC. Transverse sections of roots imaged under UV light: (C) control root, (D) treated root (Zn^2+^ + BFC) scale bar = 10 µm. (E) Fluorescence of root extracts under UV light: (E-i) control, (E-ii) Zn^2+^ only, (E-iii) BFC only, (E-iv) Zn^2+^ + BFC.

**Fig. 6:**
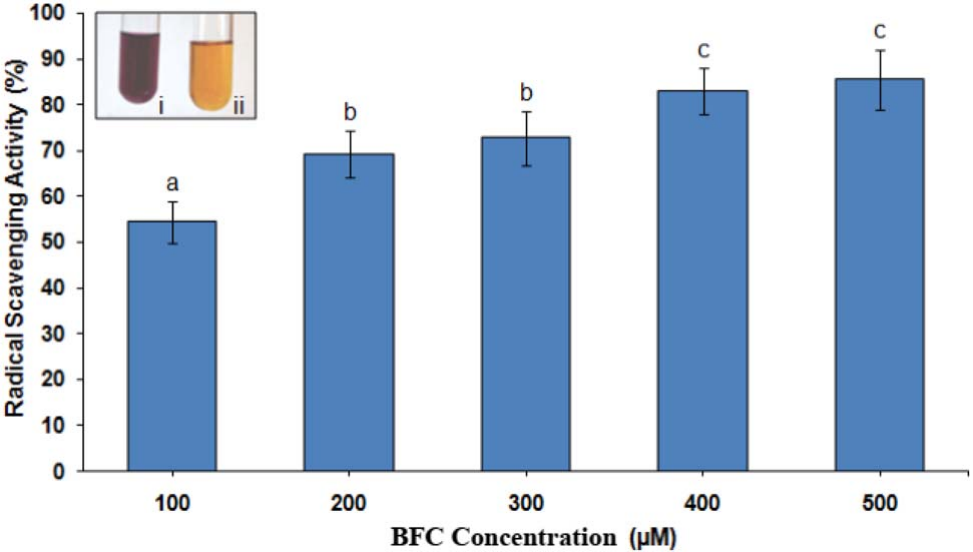
Antioxidant activity of BFC with DPPH. The results are expressed as mean ± standard deviation, and values marked with different lowercase letters indicate statistically significant differences (*p* ≤ 0.05) based on Duncan's multiple range test (*n* = 10). Inset represents the degree of color fading (purple to yellow) is proportional to antioxidant capacity, (i) only 0.1 µM DPPH, (ii) 0.1 µM DPPH + 500 µM BFC.

Antioxidants interact with DPPH by donating hydrogen atoms, thereby reducing it to DPPH-H, which results in a decrease in absorbance. The extent of this discoloration reflects the free radical scavenging capacity of the antioxidant compounds or extracts, indicating their hydrogen-donating ability. The antioxidant activity of the BFC compound was evaluated using the DPPH radical scavenging assay. As illustrated in Fig. 5, the control sample containing only the DPPH reagent retained a deep purple coloration, indicating the presence of unreacted free radicals. In contrast BFC treated samples exhibited a marked reduction in color intensity, reflecting the compound's ability to neutralize DPPH radicals through free radical scavenging. Notably, a 50% reduction in DPPH absorbance was observed at a BFC concentration of 100 µM, indicating strong antioxidant potential at relatively low concentrations. The decolorization effect appeared to be concentration dependent up to 500 µM, beyond which no substantial difference was observed between 400 µM, and 500 µM treatments (Fig. 6). This plateau suggests a saturation point in the scavenging effect. The observed transition from deep purple to lighter shades confirms the radical neutralizing capacity of BFC, highlighting its promise as a potential antioxidant agent.^43^

In this study, we demonstrated the efficacy of the fluorescent probe BFC for detecting Zn^2+^ accumulation through a plant-based cell imaging approach. *Lathyrus sativus* L. (grass pea) was selected as a low-cost, accessible, and practical model system for preliminary screening.^44,45^ Transverse sections of roots treated with BFC and Zn^2+^ exhibited distinct fluorescence, confirming the accumulation and interaction of BFC with Zn^2+^ in plant tissues (Fig. 5B and D). Additionally, when root extracts were centrifuged and the supernatant examined under UV light, strong green fluorescence was observed in BFC and Zn^2+^ treated samples. The higher fluorescent zone is observed inside stele region in treatment. This signifies that the chemical BFC absorbed by root and transmitted throughout the plant. Control samples showed no such emission (Fig. 5A and C). These findings establish BFC as a reliable fluorescent indicator for Zn^2+^ detection in plant systems. The simplicity, sensitivity, and visual clarity of this method highlight the potential of the BFC probe as a versatile tool for Zn^2+^ detection in different applications like detoxification and marker molecule for bio-absorption.

## 3. Conclusion

The present study successfully establishes BFC as a highly efficient ratiometric fluorescent probe for the selective ratiometric recognition of Zn^2+^ in semi-aqueous environments. The marked fluorescence “turn-on” response demonstrates exceptional sensitivity, with a limit of detection significantly lower than the acceptable (World Health Organisation) WHO criterion, and strong binding affinity collectively emphasize its practical significance. Comprehensive mechanistic validation through spectroscopic studies and DFT calculations further confirms the CHEF-driven sensing pathway. Importantly, the application of BFC in plant-based cell imaging highlights its ability to monitor Zn^2+^ accumulation in biological systems, demonstrating its utility beyond solution studies. Taken together, these findings underscore the potential of BFC as a simple yet powerful probe with broad applicability in environmental surveillance, food safety, and biological risk assessment, offering a reliable tool for advancing Zn^2+^ sensing technologies.

## Conflicts of interest

The authors declare no competing financial interest.

## Supplementary Material

RA-016-D5RA09973K-s001

## Data Availability

The data supporting this article have been included as part of the supplementary information (SI). Supplementary information is available. See DOI: https://doi.org/10.1039/d5ra09973k.
