# Supplementary material for: A furano–ortho–vanillin conjugate for fluorogenic ratiometric and selective Zn2+ sensing: theoretical insights and biological studies
Source: RSC Adv. 2026 Jun 1;16(32):29751–7. doi: 10.1039/d5ra09973k (PMC13227492; doi:10.1039/d5ra09973k)
Supplement: RA-016-D5RA09973K-s001 [file RA-016-D5RA09973K-s001.pdf]

Supplementary Information (SI) for RSC Advances.
This journal is © The Royal Society of Chemistry 2026

A Furano–Ortho-Vanillin Conjugate for Fluorogenic Ratiometric and Selective Zn^{2+} Sensing: Theoretical Insights and Biological Studies

Priyanka Avala[a,b], Shinziya H[a,b], Avijit Kumar Das[a,b*], Gopal Ch. Das[c], Tilak Raj Maity[d], Aveek Samanta[e], Malay Dolai[c*]

[a] *Department of Chemistry, Christ University, Hosur Road, Bangalore, Karnataka 560029, India,*
Email: avijitkumar.das@christuniversity.in, *sanjuavi.das@gmail.com*

[b] *Centre for Renewable Energy and Environmental Sustainability, Christ University, Karnataka, 560029, India*

[c]*Department of Chemistry, Prabhat Kumar College, Purba Medinipur 721404, West Bengal, India; E-mail-dolaimalay@yahoo.in*

[d] *Department of Biotechnology, Haldia Institute of Technology, Haldia, Purba Medinipur–721657, West Bengal, India*

[e] *Department of Botany, Prabhat Kumar College, Contai, Purba Medinipur 721404, W.B., India*

CONTENTS

1. **General methods of UV-*vis,* fluorescence titration experiments**
2. **Binding constant determination**
3. **Determination of fluorescence quantum yield**
4. **Calculation of the detection limit**
5. **Job plot analysis, NMR and mass spectra**
6. **Rate constant**
7. **pH effect**
8. **Comparision Table**
9. **Computational details**
10. **Cell Imaging Study**
11. **Antioxidant Assay**
12. **References**

1. Materials and Methods

1.1. General:

Chemicals, solvents including buffer solutions were procured from Sigma Aldrich. Melting points were measured using a hot-plate apparatus with open-ended capillary tubes. 1H NMR and ^13C NMR spectra were recorded on a Bruker Avance 400 MHz spectrometer using DMSO-d6 as the solvent. Chemical shifts and ^1H-^1H coupling constants are reported in δ units and Hertz (Hz), respectively. UV-visible absorption and fluorescence titration experiments were conducted using a PerkinElmer Lambda 30 UV-*vis* spectrophotometer and a Shimadzu RF-6000 spectrofluorophotometer, respectively, with a 10 mm path length fluorescence cuvette.

1.2. General method of UV-*vis* and fluorescence titration:

By UV-*vis* method:

For UV-*vis* titrations, stock solution of the sensor was prepared (c = 2.0 x 10^{-5} M) in CH_3CN-HEPES buffer (9/1, v/v, 25°C) at pH 7.4. The solution of the guest interfering analytes like Cu^{2+}, Mn^{2+}, Pb^{2+}, Cd^{2+}, Fe^{2+}, Cu^{2+}, Fe^{3+}, Zn^{2+}, Co^{2+}, Al^{3+}, Cr^{3+}, Ni^{2+} were also prepared in the order of (c =2 x 10^{-4} M). Initially sensor **BFC** solution was prepared by dissolving the sensor in 2 ml acetonitrile followed by the gradual addition of corresponding guest analytes with the particular concentration. Solutions of various concentrations containing sensor and increasing concentrations of analytes were prepared separately. The spectra of these solutions were recorded by means of UV-vis methods.

1.3 General procedure for drawing Job plot by UV–*vis* method:

Stock solution of same concentration of **BFC** and Zn^{2+} were prepared in the order of ≈ 2.0 x 10^{-5} M in CH_3CN-HEPES buffer (7:3, v/v, pH = 7.4). The absorbance in each case with different *host–guest* ratio but equal in volume was recorded. Job plots were drawn by plotting $\Delta I.X_{host}$ *vs* X_{host} (ΔI = change of intensity of the absorbance spectrum during titration and X_{host} is the mole fraction of the host in each case, respectively).

1.4 By fluorescence method:

For fluorescence titrations, stock solution of the sensor (c = 2 x 10^{-5} M) was prepared for the titration of cations in CH_3CN-HEPES buffer [9:1, v/v, pH = 7.4]. The solution of the guest cations using their chloride salts in the order of 200 μM were also prepared. Solutions of various concentrations containing sensor and increasing concentrations of cations were

prepared separately. The spectra of these solutions were recorded by means of fluorescence methods.

2. Binding constant determination:

The binding constant value of cation Zn^{2+} with the sensor has been determined from the emission intensity data following the modified Benesi–Hildebrand equation, $1/\Delta I = 1/\Delta I_{max} + (1/K[C])(1/\Delta I_{max})$. Here ΔI = I-Imin and ΔI max = Imax-Imin, where Imin, I, and Imax are the emission intensities of sensor considered in the absence of guest, at an intermediate concentration and at a concentration of complete saturation of guest where K is the binding constant and [C] is the guest concentration respectively. From the plot of (Imax-Imin)/(I-Imin) against $[C]^{-1}$ for sensor, the value of K has been determined from the slope. The binding constant (K_a) as determined by fluorescence titration method for sensor with Zn^{2+} is found to be 4×10^4 M^{-1} (error < 10%).

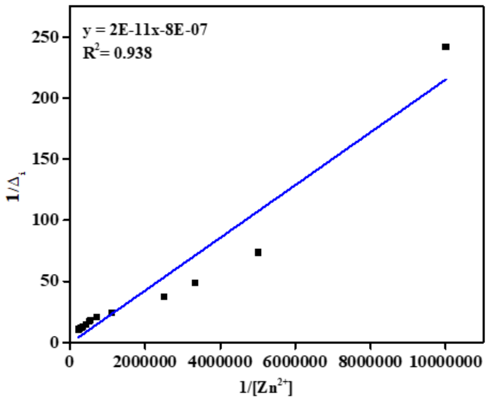

Figure S1. Benesi–Hildebrand plot from fluorescence titration data of receptor (20μM) with Zn^{2+} [G].

3. Determination of fluorescence quantum yield:

Here, the quantum yield φ was measured by using the following equation,

$$\varphi_X = \varphi_S (F_X / F_S)(A_S / A_X)(n_X^2/ n_S^2)$$

Where,

X & S indicate the unknown and standard solution respectively, φ = quantum yield,

F = area under the emission curve, A = absorbance at the excitation wave length

n = index of refraction of the solvent. Here φ measurements were performed using anthracene in ethanol as standard [φ = 0.65] (error ~ 10%)

4. Calculation of the detection limit:

The detection limit (DL) of **BFC** for $Zn^{2+}$was determined from the following equation:

$DL = K^* Sb_1/S$

Where K = 2 or 3 (we take 3 in this case); Sb_1 is the standard deviation of the blank solution; S is the slope of the calibration curve.

From the graph Fig.S1, we get slope = 19593, and Sb_1 value is 205.73

Thus, using the formula, we get the Detection Limit for Zn^{2+} = 0.65 µM.

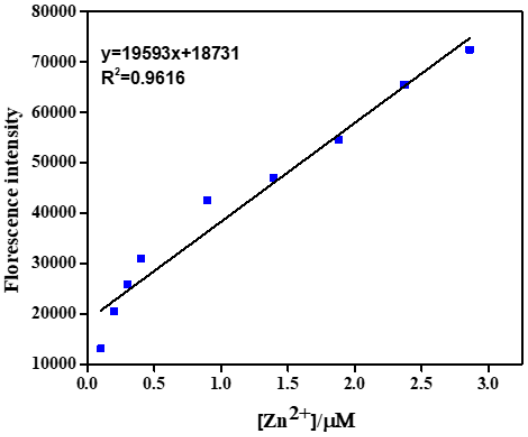

Figure S2. Changes of fluorescence intensity of **BFC** as a function of **[Zn^{2+}]**

5. Jobs plot analysis:

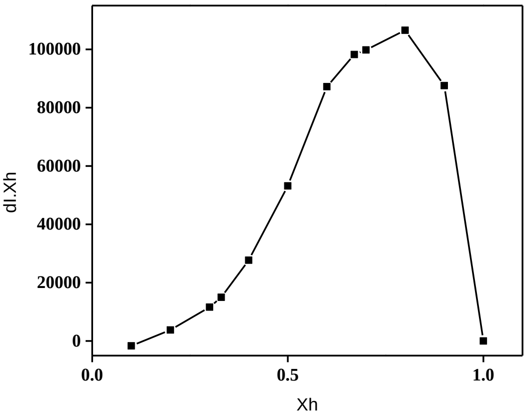

Figure S3. Job's plot diagram of receptor **BFC** for Zn^{2+} (where Xh is the mole fraction of host **BFC** and ΔI indicates the change of the intensity).

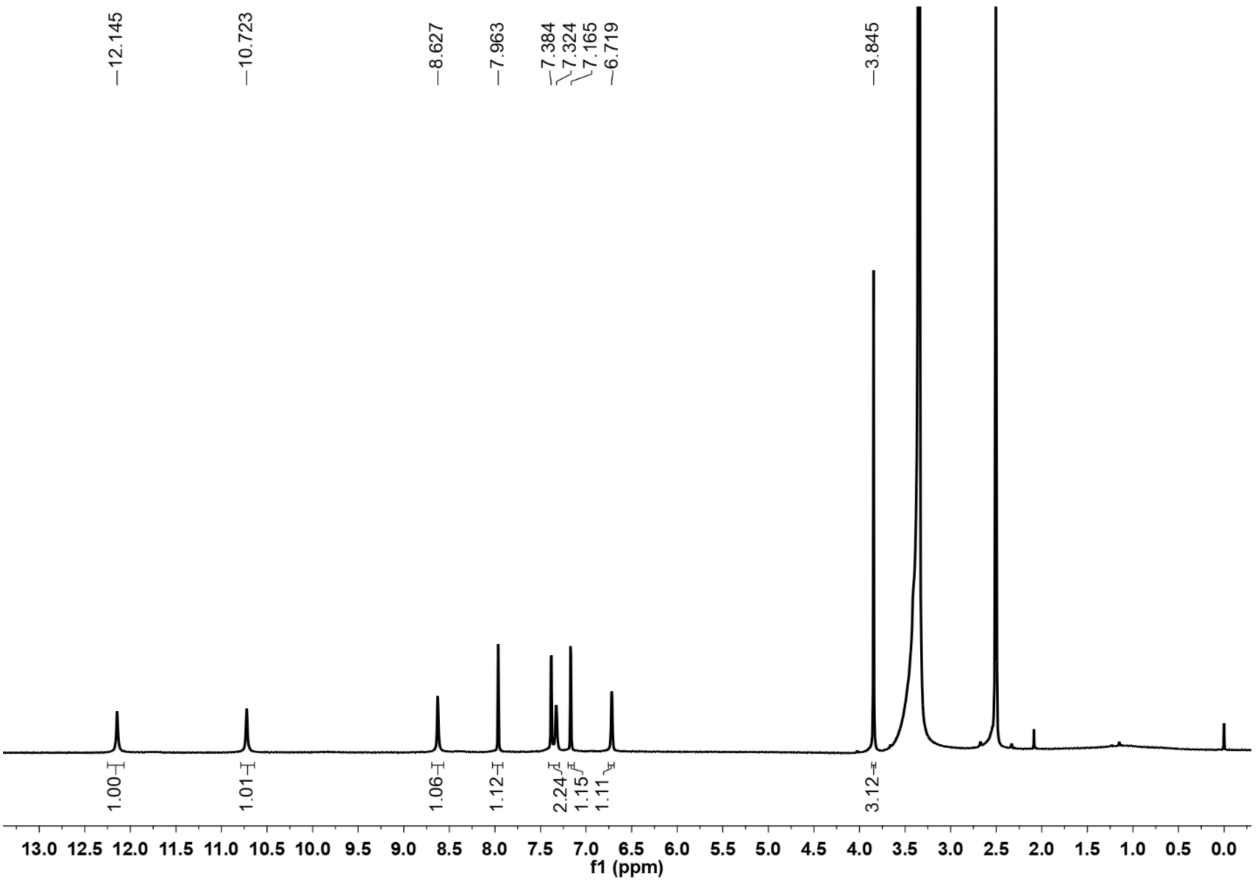

Figure S4. ^{1}H-NMR spectrum of **BFC**

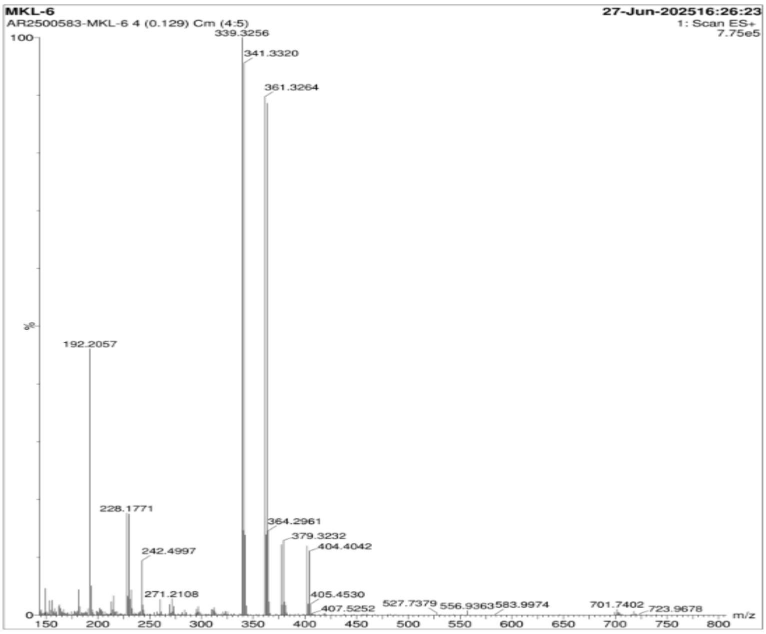

Figure S5. Mass spectrum of **BFC**

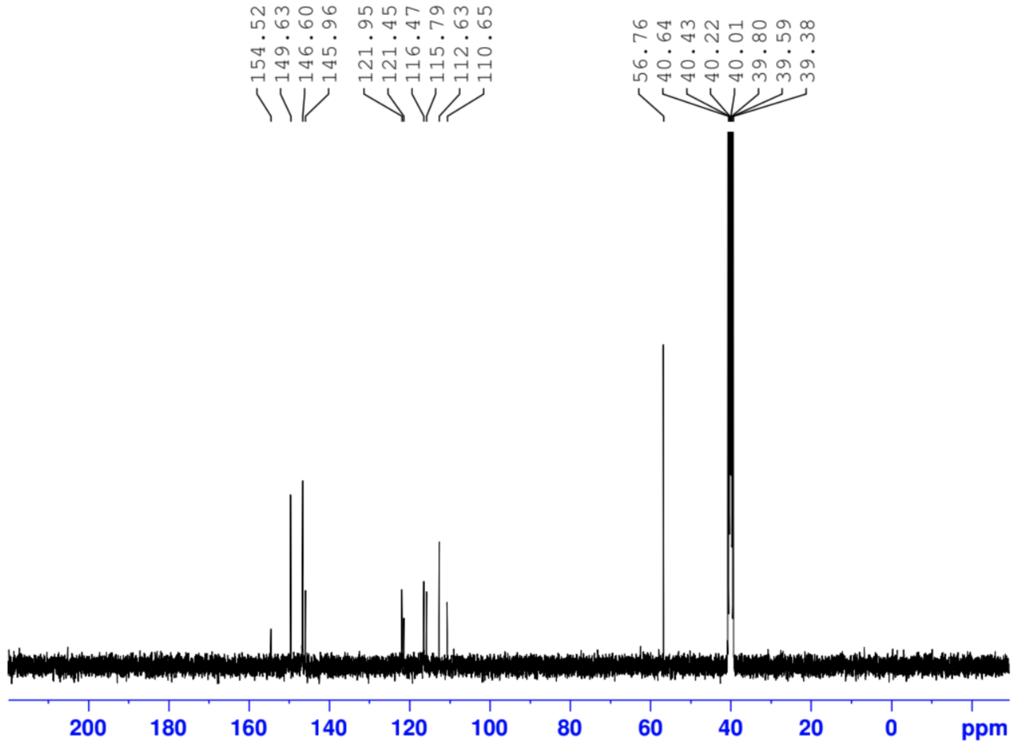

Figure S6. ^{13}C-NMR spectrum of **BFC** in DMSO-d_6

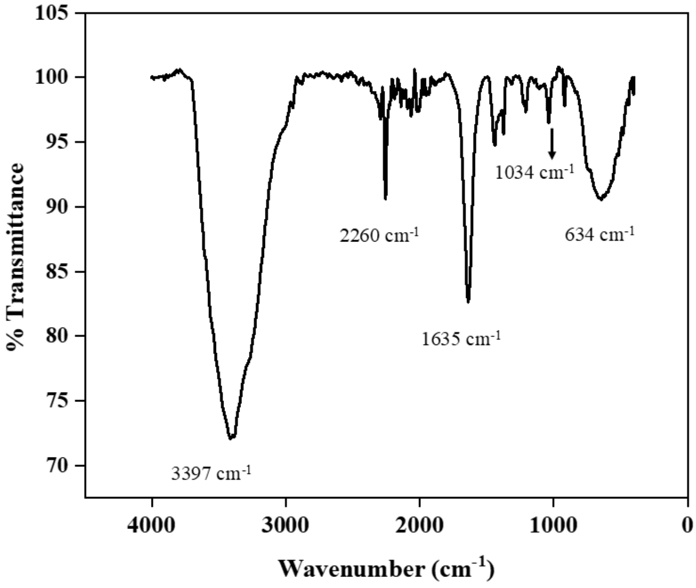

Figure S7. IR spectra of **BFC**

6. The changes of emission curve of BFC (*c* = 2.0 × 10^{-5} M) at different time interval by addition of Zinc (*c* =2× 10^{-4}) and calculation of first order rate constant:

From the time vs. fluorescent intensity plot at fixed wavelength at 490 nm by using first order rate equation we get the rate constant K = slope × 2.303 = 341.66× 2.303 = 7.86 × 10^{2} Sec^{-1}.

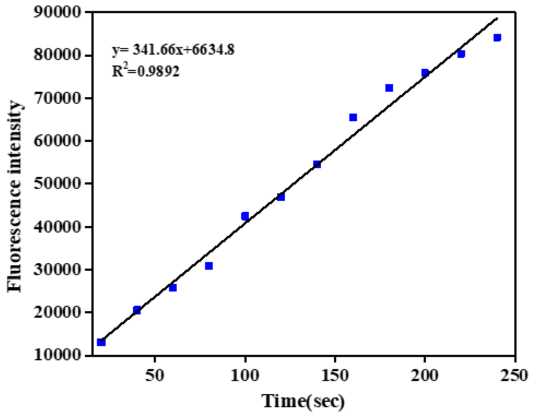

Figure S8. The first order rate equation by using Time vs. fluorescent intensity plot at 490nm.

7. pH Effect:

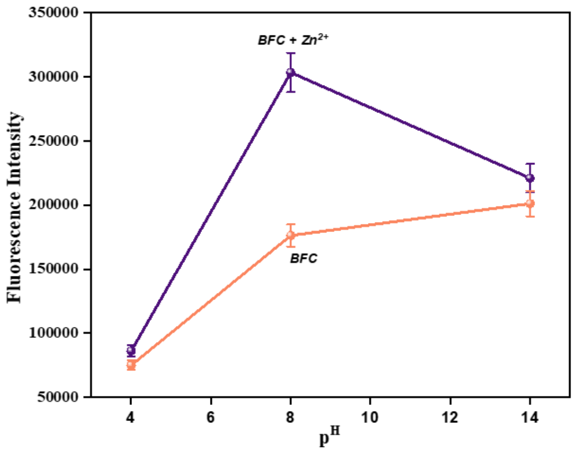

Figure S9. Fluorescence intensity of **BFC** (*c* = 2.0 x 10^{-5} M) at various pH in absence and presence of Zn^{2+} (Y error bars for both [±] deviation ranging 2%).

8.Table S1: Comparison table of BFC with the reported similar type of ligand for detection of Zn^{2+}

Ligand	Analyte	Fluorescence response	Colorimetric	LOD (µM)	Applications	Ref
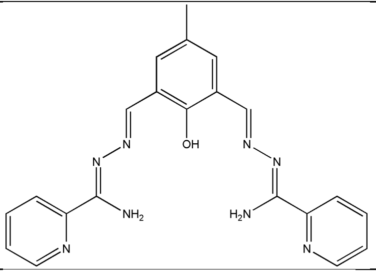	Zn^{2+}	Ratiometric	Yes	24 µM	DFT<br>Bio Imaging	[7]
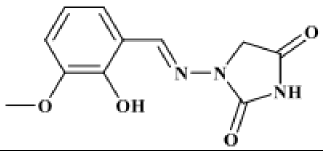	Zn^{2+}	Turn-on	Yes	11.9 µM	DFT<br>Bio Imaging	[8]
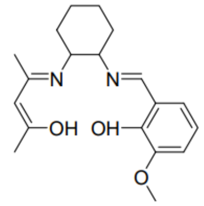	Zn^{2+}	Turn- on	No	0.09 µM	No DFT<br>No Bio imaging study	[9]
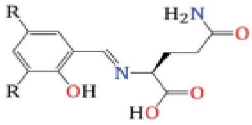<br>**A:** R = tBu<br>**B:** R = H	Zn^{2+}	Turn-on	No	1.17 µM<br>1.2 µM	DFT<br>Living cells	[10]
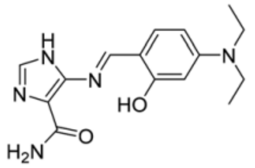	Zn^{2+}	Turn-on	Yes	1.59 µM	DFT<br>Living cells	[11]
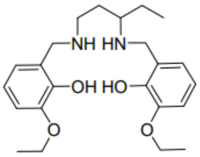	Zn^{2+}	Turn-on	No	0.774 µM	No DFT<br>No Bio Imaging study	[12]
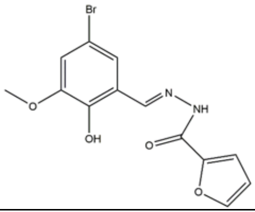	Zn^{2+}	Ratiometric	yes	0.65 µM	DFT<br>Antioxidant Assay and Bio Imaging study	[This work]

9. Computational details

Density Functional Theory (DFT)[1] calculations were conducted using the Gaussian 09 (Revision A.02) package, with "Gauss View" utilized for visualizing molecular orbitals. Becke's three-parameter hybrid-exchange functional, the Lee-Yang-Parr expression for nonlocal correlation, and the Vosko-Wilk-Nuair 1980 local correlation functional (B3LYP) were employed in the calculation.[2] Optimization of **BFC** and **BFC-Zn^{2+}** in the gas phase were performed using the 6-31+(g) basis set. The absorbance spectral properties of **BFC** and **BFC-Zn^{2+}** were calculated by time-dependent density functional theory (TDDFT)[3] associated with the conductor-like polarizable continuum model and we computed the lowest 40 singlet – singlet transition. For H, C, N, O, atoms we used 6-31+(g) basis set for all the calculations. The calculated electron-density plots for frontier molecular orbitals were prepared by using Gauss View 5.1 software. All the calculations were performed with the Gaussian 09W software package.[4]

10.Cell imaging study

Grass pea (*Lathyrus sativus* L.) seeds were surface sterilized with 1% bavistin followed by 0.1% $HgCl_2$ for 5 min and washed in distilled water (3 times, 10 min each). Washed seeds were soaked in distilled water and allowed to germinate in cotton bed for 2 days. The germinated seedlings were then treated with aqueous solutions of 1 mM Zinc sulfate ($ZnSO_4$) for 2 days and followed by 10 μM of **BFC** for 2 days. Sterilized seeds treated with distilled water were marked as control. On 6th day roots from different treatments were washed with distilled water and crushed with 0.1 M potassium phosphate buffer of pH 7 using mortar and pestle. The homogenate was centrifuged (Remi C-24 plus) at room temperature for 10 min at 7,000 rpm and the supernatant was observed the fluorescence under UV light. Transverse section of roots observed under UV light using Zeiss fluorescent microscope.[5,6]

11.Antioxidant Assay

The radical scavenging activity of **BFC** was determined by using DPPH (2,2-diphenyl-1-picrylhydrazyl) assay according to Baliyan et al. (2022). The decrease in the absorption of the DPPH solution after the addition of an antioxidant was measured at 517 nm. 0.1 mM DPPH solution was prepared by dissolving 2 mg DPPH in 50 ml of ethanol. Different concentrations of **BFC** (100 μM, 200 μM, 300 μM, 400 μM and 500 μM) were added with 0.1 mM DPPH. The reaction mixture was incubated in dark at room temperature for 30 min. After 30 min, the absorbance of the mixture was read at 517 nm. 3 ml of DPPH was taken as control, where

ethanol treated as blank. The % radical scavenging activity of **BFC** was calculated using the following formula,

% RSA = [(Abs control - Abs sample) / Abs control] * 100

[Where, RSA is the Radical Scavenging Activity; Abs control is the absorbance of DPPH + ethanol; Abs sample is the absorbance of DPPH + BFC].

12.REFERENCES:-

[1] R. G. Parr,S. R. Gadre andL. J. Bartolotti,*Local density functional theory of atoms and molecules*,Proc. Natl. Acad. Sci. U.S.A., 1979, **76**, 2522–2526.

[2] (a) A. D. Becke, *Density-functional thermochemistry. III. The role of exact exchange*, J. Chem. Phys.**,** 1993, **98**, 5648–5652 (b) C. Lee, *Effective homogeneity of the exchange–correlation energy functional*, Phys. Rev. B, 1998

[3] R. Bauernschmitt, R. Ahlrichs, Treatment of electronic excitations within the adiabatic approximation of time dependent density functional theory, Chem. Phys. Lett. 1996,**256**,454–464.

[4] M. J. Frisch, G. W. Trucks, H. B. Schlegel, G. E. Scuseria, M. A. Robb, J. R. Cheeseman, G. Scalmani, V. Barone, B. Mennucci, G. Petersson and H. Nakatsuji, *Gaussian*, 2009,**227,**9(9)

[5] S. Saha, S. Das and P. Sahoo, *ChemistrySelect*, 2019, **4**, 13968–13973.

[6] A. Samanta, S. Banerjee, T. R. Maity, J. Jahnavi and S. Datta, *Protoplasma*, 2022, **259**, 1455–1466.

[7] K. Kumarasamy, Z.-W. Wu, W.-J. Chien, M.-C. Lin and S. K. Ramasamy, *J. Environ. Chem. Eng.*, 2024, **12**, 114615.

[8] M. S. Kim, T. G. Jo, M. Yang, J. Han, M. H. Lim and C. Kim, *Spectrochim. Acta A Mol. Biomol. Spectrosc.*, 2019, **211**, 34–43.

[9] L.-Y. Shen, X.-L. Chen, X.-J. Yang, H. Xu, Y.-L. Huang, X. Zhang, C. Redshaw and Q.-L. Zhang, *Molecules*, 2021, **26**, 3825.

[10] J. C. Berrones-Reyes, B. M. Muñoz-Flores, A. M. Cantón-Diáz, M. A. Treto-Suárez, D. Páez-Hernández, E. Schott, X. Zarate and V. M. Jiménez-Pérez, *RSC Adv.*, 2019, **9**, 30778–30789.

[11] J. Y. Yun, J. B. Chae, M. Kim, M. H. Lim and C. Kim, *Photochem. Photobiol. Sci.*, 2019, **18**, 166–176

[12] M. Karmakar and S. Chattopadhyay, *Polyhedron*, 2020, **187**, 114639.
